# Co-infection of two eukaryotic pathogens within clam populations in Arcachon Bay

**DOI:** 10.3389/fmicb.2023.1250947

**Published:** 2024-01-08

**Authors:** Sarah Itoïz, Clara Mouronvalle, Morgan Perennou, Elisa Chailler, Morgan Smits, Evelyne Derelle, Sebastian Metz, Nelly Le Goïc, Adeline Bidault, Xavier de Montaudouin, Isabelle Arzul, Philippe Soudant, Aurélie Chambouvet

**Affiliations:** ^1^Univ Brest, CNRS, IRD, Ifremer, LEMAR, Plouzané, France; ^2^EPHE, PSL Research University, UPVD, CNRS, USR CRIOBE, Perpignan, France; ^3^CNRS, UMR7144 Adaptation et Diversité en Milieu Marin, Ecology of Marine Plankton (ECOMAP), Station Biologique de Roscoff SBR, Sorbonne University, Roscoff, France; ^4^Univ. Bordeaux, CNRS, Bordeaux INP, EPOC, UMR, Station Marine d’Arcachon, Arcachon, France; ^5^Ifremer, ASIM Adaptation et Santé des Invertébrés Marins, La Tremblade, France

**Keywords:** Perkinsosis, parasite, multiple-infection, co-infection, Manila clam, organ distribution

## Abstract

The parasitic species *Perkinsus olseni* (= *atlanticus*) (Perkinsea, Alveolata) infects a wide range of mollusc species and is responsible for mortality events and economic losses in the aquaculture industry and fisheries worldwide. Thus far, most studies conducted in this field have approached the problem from a “one parasite-one disease” perspective, notably with regards to commercially relevant clam species, while the impact of other *Perkinsus* species should also be considered as it could play a key role in the disease phenotype and dynamics. Co-infection of *P. olseni* and *P. chesapeaki* has already been sporadically described in Manila clam populations in Europe. Here, we describe for the first time the parasitic distribution of two *Perkinsus* species, *P. olseni* and *P. chesapeaki*, in individual clam organs and in five different locations across Arcachon Bay (France), using simultaneous *in situ* detection by quantitative PCR (qPCR) duplex methodology. We show that *P. olseni* single-infection largely dominated prevalence (46–84%) with high intensities of infection (7.2 to 8.5 log-nb of copies. g^−1^of wet tissue of Manila clam) depending on location, suggesting that infection is driven by the abiotic characteristics of stations and physiological states of the host. Conversely, single *P. chesapeaki* infections were observed in only two sampling stations, Ile aux Oiseaux and Gujan, with low prevalences 2 and 14%, respectively. Interestingly, the co-infection by both *Perkinsus* spp., ranging in prevalence from 12 to 34%, was distributed across four stations of Arcachon Bay, and was detected in one or two organs maximum. Within these co-infected organs, *P. olseni* largely dominated the global parasitic load. Hence, the co-infection dynamics between *P. olseni* and *P. chesapeaki* may rely on a facilitating role of *P. olseni* in developing a primary infection which in turn may help *P. chesapeaki* infect *R. philippinarum* as a reservoir for a preferred host. This ecological study demonstrates that the detection and quantification of both parasitic species, *P. olseni* and *P. chesapeaki*, is essential and timely in resolving cryptic infections and their consequences on individual hosts and clam populations.

## 1 Introduction

Co-infection by multiple parasites has become the rule rather than the exception since the revision of the gold-standard – but outdated – paradigm “one parasite-one disease” (Petney and Andrews, 1998). Deciphering these interactions is essential for understanding the dynamics and phenotypic outcomes of diseases (Paul-Pont et al., 2010; Vayssier-Taussat et al., 2014; Bass et al., 2019). Indeed, the prediction of co-infection outcomes is tricky because heterogeneous disease phenotypes can emerge with the amplification or the suppression of the weight of one or more parasite species (Johnson and Hoverman, 2012) by altering direct competition for resources or space (ecological interactions) or infectivity towards the host immune system (immunological interactions) (Cox, 2001; Graham, 2008). In medical and veterinary fields, co-occurrence of multiple pathogens has gained significant interest with reported studies on human (e.g., between different *Plasmodium* spp.: see McKenzie and Bossert, 1999) and animal health (e.g., multiple *Borrelia* species in ticks: Raileanu et al., 2017). However, in the field of marine protistology and microbial parasites, this concept represents a Pandora’s box that needs to be opened. One explanation for this lasting knowledge gap is the difficulty inherent in characterising these micro-parasitic organisms, which has often been limited to culture and microscopy techniques. The advent of molecular biology and environmental sequencing has revolutionised the concept of microbial diversity with the (re)-discovery and (re)-characterisation of many eukaryotic parasite species (Moon-van der Staay et al., 2001; López-García et al., 2003; Chambouvet et al., 2008). This has brought new insights in species definition and unravelled many cases of cryptic infectious agents (Putaporntip et al., 2009; Chambouvet et al., 2015), but the prevalence and severity of mortality events associated with these pathogens continue to increase worldwide due to global change, making it all the more urgent to tackle the fundamental questions regarding the role of multiple infections in the dynamics of aquatic disease in marine ecosystems (Harvell, 1999; Burge et al., 2014).

The marine parasite *Perkinsus olseni* (Perkinsea, Alveolata) infects commercially important bivalve (e.g., clams and oysters) and gastropod species (abalone) and is the aetiological agent of Perkinsosis disease (see review by Ruano et al., 2015). Heavily infected hosts might show the presence of milky white nodules in the gills, mantle, and foot, a disruption of connective tissue and epithelial cells, pale appearance of the digestive gland, reduction of the condition index, and severe emaciation, all of which could contribute to host death (Ruano et al., 2015). In Europe, *P. olseni* is regularly detected along the Atlantic and Mediterranean coasts and has been associated with high mortality rates, resulting in severe economic loss mainly in Portugal, Spain, and along the Mediterranean coast (Da Ros and Canzonier, 1985; Ruano and Cachola, 1986; Azevedo, 1989; Pretto et al., 2014). It has been hypothesised that *P. olseni* was accidentally co-introduced into European waters with its host the Manila clam (*Ruditapes philippinarum*), which was imported from Asia in the 1970s for aquaculture purposes (de Montaudouin et al., 2016a). This hypothesis is supported by the low genetic diversity observed in *P. olseni* in Europe compared to those collected in Japan and in New Zealand (Vilas et al., 2011). However, the situation may not be so clear-cut given recent findings that revealed *P. olseni* is not the only *Perkinsus* parasite detected in Europe. Indeed, along the Atlantic coast, this parasite co-infects in sympatry with a congeneric species, *P. chesapeaki*, in *Ruditapes decussatus* from Leucate lagoon, France (Arzul et al., 2012) and Manila clams, *R. philippinarum*, from Arcachon Bay (France) (Itoïz et al., 2021) and in Galicia (Spain) (Ramilo et al., 2016). The parasite *P. chesapeaki* was firstly detected in the soft-shell clam *Mya arenaria* from the Chesapeake Bay, U.S.A. (McLaughlin and Faisal, 2000). Since then, it has been detected in many other bivalves, e.g., *Mercenaria mercenaria* (Reece et al., 2008), *Tagelus plebeius* (Bushek et al., 2008) or *Crassostrea rhizophorae* (Dantas Neto et al., 2016), in South America, North America, Asia, and Europe (see review by Itoïz et al., 2022). This parasitic species has also been found in association with *Perkinsus marinus* in oysters and clams from the Chesapeake Bay (U.S.A.) (Reece et al., 2008). One hypothesis suggests that, much like *P. olseni*, *P. chesapeaki* was putatively and accidentally introduced to Europe through its hosts the soft-shell clam, *Mya arenaria*, or the hard clam, *Mercenaria mercenaria* from the U.S.A (Arzul et al., 2012). While no mortality event has thus far been associated with *P. chesapeaki* (e.g., Bushek et al., 2008; Carrasco et al., 2014), co-infection in sympatry by these congeneric species, *P. olseni* and *P. chesapeaki*, remains enigmatic according to the competitive exclusion principle (Gause, 1934) and warrants further explanations. Furthermore, *Perkinsus* species are not the only pathogens present in Arcachon Bay. Manila clams may also become infected by a virus-like agent Brown Muscle Disease (BMD) (Dang et al., 2008) and by *Vibrio tapetis,* the bacterial agent of Brown Ring Disease (BRD) (Paillard, 2004). Although these two infectious agents were regularly detected in Arcachon Bay, their prevalence remains quite low compared to those of *Perkinsus* spp. (de Montaudouin et al., 2010).

The aim of this study was to evaluate (1) the distribution of *P. olseni* and *P. chesapeaki* parasites across five contrasted sampling areas in Arcachon Bay, a lagoon where *Perkinsus* spp. is particularly prevalent and abundant (Dang et al., 2008; de Montaudouin et al., 2010); and (2) the occurrence of these two parasitic protists across each organ type of individual clams from the five sampling stations.

## 2 Materials and methods

### 2.1 Sampling site and strategy

**Figure 1:**
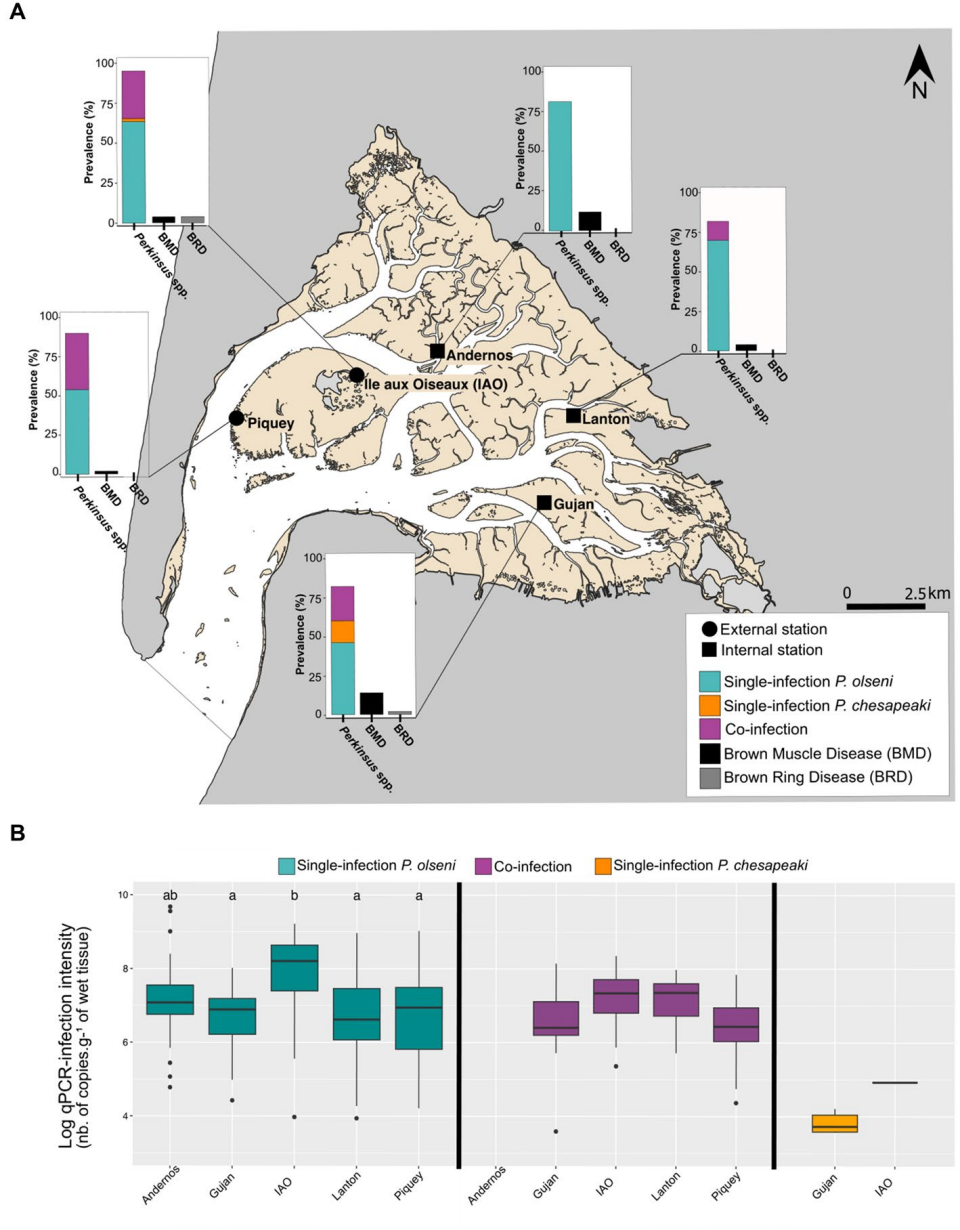
Distribution of infection and co-infection in whole clam body from five stations sampled in November 2018 in the Arcachon bay. **(A)** Prevalences of *P. olseni* single-infection, *P. chesapeaki* single-infection and (co-)infections determined by duplex qPCR with prevalences of Brown muscle disease (BMD) and Brown ring disease (BRD). Each prevalence was determined on a total of 50 clams per station. **(B)** Log-qPCR infection intensities estimated on the whole-body clam are represented for each type of infection: *P. olseni* single-infection, *P. chesapeaki* single-infection and co-infection. Differences are estimated by a *pairwise Wilcoxon* rank sum *test.* Differences between each parameter are represented by a and b.

Arcachon Bay (South West of France, Atlantic coast, 44°41′60” N; 1°10’W) is a mesotidal semi-sheltered lagoon of 180 km^2^. This marine system is under oceanic (Atlantic tidal regime) and freshwater influences (Leyre River flow) encompassed by a 280 km^2^ drainage basin. The exotic Manila clam, *Ruditapes philippinarum* was introduced in France in 1972 for aquaculture purpose (de Montaudouin et al., 2016a). Since then, Arcachon Bay clam populations ranked first in France in terms of biomass and exploitation, despite a significant lack of spat recruitment and growth (de Montaudouin et al., 2016b). Perkinsosis disease is regularly detected in local Manila clam populations in Arcachon Bay, but no associated mortality events have been reported so far (Dang et al., 2010a,b; de Montaudouin et al., 2016b). Sampling station localisation within the lagoon and some environmental parameters were determined for Andernos station exclusively by de Montaudouin et al. (2010) and at a higher scale by Binias et al. (2014). Here, we sampled 50 Manila clams on the 7th to 8th of November 2018 in five contrasted stations for which some environmental data were available (Dang et al., 2010a; Binias et al., 2014; Figure 1). Two stations (IAO and Piquey) are in “group 2” as defined by Binias et al. (2014), and qualified herein as “external.” This group gathered stations in oceanic position with high grain size median, high mean salinity, low organic matter and silt & clay contents (Supplementary Table S1). Piquey exhibits higher sediment grain size median than IAO. Andernos, Lanton and Gujan belong to “group 4” as defined by Binias et al. (2014), and qualified herein as “internal.” This group gathered stations undergoing lower mean salinity. In this group, Andernos differs from Gujan and Lanton by its coarser sediments (Table 1). All these stations are situated at a rather high tidal level (Table 1), and Piquey is the only one close to oyster parks and oyster reefs.

**Table 1:** Prevalence of *Perkinsus* spp., *Perkinsus olseni* and *Perkinsus chesapeaki* in gill tissue samples for each sampling site.

Site	*n*	*Perkinsus* spp. prevalence (RFTM assay)	*Perkinsus* spp. prevalence (qPCR assay)	*P. olseni* prevalence (qPCR assay)	*P. chesapeaki* prevalence (qPCR assay)
Andernos	50	90	66	66	0
Gujan	50	86	62	62	0
IAO	50	89	90	90	6
Lanton	50	74	72	72	6
Piquey	50	98	82	82	24

The methodology used to assess the prevalence is in bracket. n: total number of Manila clams sampled. Prevalences are in ‘%’.

#### 2.1.1 Manila clam biometry

The shell length of all clams was measured individually using a vernier calliper. Normality, homogeneity of variance (Shapiro–Wilk normality test) and one-way analysis of variance (ANOVA) were conducted in order to compare size distribution between sites, before multiple comparisons of means by Tukey test.

#### 2.1.2 Sample preparation

Each clam was dissected on ice and each organ was weighted independently. For each individual, one gill was incubated in RFTM (Ray’s Fluid Thioglycolate Medium) supplemented with antibiotics (penicillin G, 1x10^5^U.L^−1^ and streptomycin sulphate 0.1 g.L^−1^, Sigma) and antimycotic (nystatin 4 g.L^−1^) for *Perkinsus* spp. cells counting. The six other organs, including the second gill, the digestive gland, the mantle, the adductor muscle, the foot and the remaining tissue were fixed separately in 80% ethanol and stored at 4°C for molecular analysis.

#### 2.1.3 Visual inspection of BRD and BMD infectious agents

During the dissection, other disease phenotypes associated with commonly detected infectious agents in Manila clams such as Brown Muscle Disease (BMD) or the Brown Ring Disease (BRD) were considered. Briefly, we checked the presence of necropsies of the posterior adductor muscle for the virus-like agent of BMD (Dang et al., 2008; Pierron et al., 2019) and the presence of a brown organic conchiolin deposit in the inner face of the valves for the *Vibrio tapetis* agent of BRD (see Paillard et al., 2006). Prevalence (in %) for both diseases was determined for each sampling station. Their distributions between stations were tested by Fisher’s exact test.

#### 2.1.4 *Perkinsus* spp. intensity of infection in gill tissue samples

Infection intensity in gill tissue was determined using the Ray’s Fluid Thioglycollate Medium (RFTM) as recommended by the O.I.E. (Ray, 1952; Choi et al., 1989; OIE-Listed Diseases 2021: OIE – World Organisation for Animal Health, 2021). Briefly, gills were incubated in RFTM medium supplemented with antibiotics (penicillin G, 1×10^5^ U.L^−1^ and streptomycin sulphate 0.1 g.L^−1^, Sigma) for 5 days in the dark at room temperature. This step allows for the transformation of trophozoites into enlarged and more visible hypnospores (Ray, 1952). To lyse the gill tissue, the RFTM was discarded following a 1,000 × *g* centrifugation and the remaining gills were digested with 2 M NaOH solution for 3 h at 60°C preserving the cell structure of *Perkinsus* hypnospores (Choi et al., 1989). Hypnospores were then recovered using 1,000 × *g* centrifugation for 10 min. The remaining pellet was washed twice in PBS and stained with a Lugol’s iodine solution (4%). The number of *Perkinsus* hypnospores per individual gill was assessed using an aliquot of 100 μL in a Nageotte chamber (10 lines counted in triplicate) under an optical microscope (Leica DM-IRB; x10 magnification). The counting results were expressed as the number of hypnospores per gram of wet tissue.

### 2.2 DNA extraction of clam tissue samples

The genomic DNA (gDNA) of the six organs (gill, digestive gland, mantle, adductor muscle, foot and the remaining tissue samples) was extracted using the CTAB-based DNA extraction method adapted from Winnepenninckx et al. (1993) described by Itoïz et al., 2021. Clam tissue samples were transferred in bead beating tubes containing three different sizes of beads (2.8 mm, 1.4 mm and 0.1 mm of diameter, Ozyme) with 1 mL of CTAB extraction buffer (2% CTAB, 100 mM TrisHCl pH = 8.0, 20 mM EDTA, 1.4 mM NaCl). Tissues were ground and homogenised following two bead beating cycles (45 s of bead beating at 6 m.s^−1^ followed by 20 s stop) in a cooling rack of the FastPrep-24 5G benchtop homogeniser (MP Biomedicals). β-mercaptoethanol (0.2%) and proteinase K (1 g.L^−1^) were then added to each tube and samples were incubated for 30 min at 60°C. Foot tissue samples were specifically incubated 12 h due to the tougher tissue structure. Lysates were mixed in chloroform/isoamylalcohol (24:1, v/v) and emulsified, then centrifuged at 18,000 × *g* for 10 min at 4°C. This step is repeated twice for foot tissue samples. Aqueous phases were treated with RNAse solution (10 g.L^−1^) (Sigma-Aldrich) for 30 min at 37°C prior to DNA precipitation with cold isopropanol overnight at 4°C. DNA was pelleted, rinsed twice with cold 70% ethanol, dried at room temperature and resuspended in 300 μL of pure molecular grade water (Corning). gDNA samples were quantified using the Qubit dsDNA HS assay kit (Invitrogen) and stored at −20°C until further processing.

### 2.3 Real-time qPCR duplex diagnostic assay for *Perkinsus olseni* and *Perkinsus chesapeaki*

Real-time qPCR duplex assays were carried out as described by Itoïz et al. (2021). Briefly, *P. olseni* and *P. chesapeaki* plasmidic-standards from 2.5×10^1^ to 2.5×10^6^ total copy number were used to calculate infection intensity. This standard-plasmidic material was previously used in Itoïz et al. (2021) allowing for identical qPCR parameters in this study (*P. olseni*: *y* = −3.33x + 39.49, efficiency (E) = 99.8%, limit of detection (LOD) = 2.5×10^1^ total copies; *P. chesapeaki*: *y* = −3.38x + 40.29, *E* = 97.8%, LOD = 2.5×10^1^ total copies).

Each organ gDNA was diluted following specific concentrations recommended in Itoïz et al. (2021) to avoid PCR inhibitors. Gill and digestive gland tissue samples were diluted to 20 ng.μL^−1^; adductor muscle and foot to 5 ng.μL^−1^; and, mantle and remaining tissue to 2 ng.μL^−1^. Real-time PCR was performed using the LightCycler 480 II (Roche) thermocycler using LightCycler 480 Probes Master (Roche) kit following the manufacturer’s recommendations. All probes and primers used in this study were described in Itoïz et al. (2021). Briefly, the reaction volume for duplex qPCR contained 1X LightCycler 480 Probes Master premixed, 0.2 μM of primer PolsITS2-F, 0.2 μM of PolsITS2-R, 0.2 μM of PchesITS2-F, 0.2 μM of PchesITS2-R, 0.5 μM of PolsITS2-probe (FAM), 0.5 μM of PchesITS2-probe (LC640), and 5 μL of DNA sample with adjusted concentration. The reaction volume for simplex qPCR contained 1X LightCycler 480 Probes Master premixed, 0.2 μM of primer PolsITS2-F or PchesITS2-F, 0.2 μM of PolsITS2-R or PchesITS2-R, 0.5 μM of PolsITS2-probe or PchesITS2-probe, 5 μL of DNA sample with adjusted concentration. The thermal cycling conditions were described in Itoïz et al. (2021). In each qPCR run, diluted gDNA samples, plasmidic-standards and two negative controls (non-infected Manila clam DNA and pure molecular grade water) were run. Ct value triplicates were averaged for downstream statistical analysis. If one or two of the triplicates were outliers, the sample was reprocessed to eliminate potential manipulation error. Each gDNA per organ was processed independently, allowing an estimation of concentrations of both parasites per organ. The whole-body concentration was calculated by summing the parasite concentrations for all organs per clam.

### 2.4 Statistical analysis of prevalence and infection intensities

#### 2.4.1 Comparison of RFTM and qPCR methodologies on gill tissue samples

To confirm congruence between RFTM and qPCR methodologies, gill tissue samples were chosen to calculate the indicative parameters of prevalence, concordance, and infection intensity following the O.I.E. recommendations for *Perkinsus* spp. diagnosis (OIE-Listed Diseases 2021: OIE – World Organisation for Animal Health, 2021). *Perkinsus* prevalence (% of infected individuals out of the total number of hosts sampled) were plotted for both methods and single-or co-infection was represented when possible. The concordance and the discordance parameters, adapted from Langton et al. (2002), were calculated. The concordance, calculated by counting paired positive samples and paired negative samples, is the percentage of chance that an identical sample analysed by two different methodologies will yield the same result. The discordance, calculated by counting the unpaired samples (i.e., positive in RFTM and negative in qPCR assay, and vice versa), is the percentage of chance that an identical sample analysed by two different methodologies will yield different results. The Cohen’s Kappa (κ) coefficient was estimated to compare the level of agreement obtained between the real-time PCR and the RFTM assays (Pfeiffer, 2010). Finally, the relationship between both methods was tested using a linear model and the Spearman correlation coefficient on infection intensities established by qPCR and RFTM from paired-positive individuals.

The global prevalence and infection intensities of the *Perkinsus* genus were calculated on gill samples using standard RFTM methodology. The infection intensity means (nb of hypnospores. g^−1^ of wet gill) measured by the two methods were compared to each other by a non-parametric Kruskal-Wallis test followed by Dunn post-hoc test.

#### 2.4.2 Distribution and infection intensity of two *Perkinsus* species in host tissue samples

Infection intensity, prevalence and tissue distribution of *P. olseni* and *P. chesapeaki* were estimated using the duplex qPCR methodology across six Manila clam organs: gills, digestive gland, adductor muscle, foot, mantle and the remaining tissue. Prevalences in each station were compared by Fisher’s exact test, adapted for small samples. Infection intensity means were evaluated in each station for each type of infection (*P. olseni* single-infection, *P. chesapeaki* single-infection, co-infection) and compared by a pairwise Wilcoxon rank sum test.

The distribution of single-and co-infected individuals in the Arcachon Bay was investigated using multivariate analysis on *P. olseni* and *P. chesapeaki* infection intensities in each organ. A principal component analysis (PCA) was performed using the FactomineR and Factoextra packages on R (Lê et al., 2008; Kassambara and Mundt, 2017). Extracted coordinates (axis 1; 2) of each individual were checked for significant differences among stations by non-parametric Kruskal-Wallis test in combination with multiple pairwise comparison using Dunn post-hoc test.

Repartition of both *Perkinsus* species across co-infected host organs (gills, digestive gland, adductor muscle, foot, mantle and the remaining tissue) was detailed in a visual matrix table. Global infection intensities (copy number.g^−1^ of wet tissue) for *P. olseni* and *P. chesapeaki* were log-transformed and classified in four levels represented with a colour gradient. Infection intensity data follows a Gaussian distribution, thus, the boundaries of the four categories were delimited by: minimum, 1st quartile, median, 3rd quartile and maximum values of infection intensity. Mean occupation of *P. olseni* and *P. chesapeaki* cells (in %) was represented for each type of co-infected organ sample from each station. Finally, *P. olseni* and *P. chesapeaki* log-transformed infection intensities from co-infected organs were plotted to investigate potential negative or positive influence of one species on the other and vice versa. Based on a reduced dataset restricted to co-infected organ samples, the relationship was tested using a linear model and Spearman correlation coefficient.

## 3 Results

### 3.1 Comparison of RFTM and qPCR assays for the detection of *Perkinsus* spp. in gills

The duplex qPCR assay developed by Itoïz et al. (2021) allowed simultaneous detection and quantification of both parasitic species, *P. olseni* and *P. chesapeaki* in the Manila clam (Table 1). We estimated the concordance and correlation between RFTM and duplex qPCR assays on gill tissue samples across all sampling stations (Supplementary Table S2). To guarantee an accurate representation of the qPCR detection, only Ct values within the standard range from 2.5 × 10^1^ to 2.5 × 10^6^ total copy number were considered as positive, as recommended by Itoïz et al. (2021).

Using RFTM and qPCR assays in gill tissue samples, the overall prevalence of *Perkinsus* spp. showed no significant differences between sampling stations (Chi-squared test: *X*^2^ = 4.14, df = 4, value of *p* > 0.05). Using RFTM assays, the prevalence of *Perkinsus* spp. ranged from 74% (Lanton) to 98% (Piquey) (Table 1). Using qPCR assays, prevalence for both *Perkinsus* species ranged from 62% (Gujan) to 90% (IAO). Concordances between RFTM and qPCR methodologies across all stations varied from 76% (Andernos and Gujan) to 86% (IAO and Lanton) (Supplementary Table S2). Overall, the mean concordance was 81.6 ± 5.2% (*n* = 5 stations).

Additionally, the *κ*-value was calculated to determine the level of agreement between each methodology for the gill samples (Supplementary Table S2). The *κ* values ranged from a slight agreement, 0.17 for Piquey, through moderate agreement, 0.35, 0.41 and 0.42 for Andernos, IAO and Gujan respectively, to substantial agreement, 0.62 for Lanton. The overall *κ*-value for all sampling stations was 0.42 indicating moderate agreement.

A linear regression was determined between for the qPCR-infection intensity and the RFTM-infection intensity (*y* = 0.56x + 4.34, y: log of qPCR-infection intensity in copy number.g of wet gill^−1^; x: log of RFTM-infection intensity in nb of cells.g of wet gill^−1^; adjusted-*R*^2^ = 0.41; *n* = 178) (Supplementary Figure S1). A significant positive correlation was determined using Pearson’s coefficient between the qPCR-infection intensity and the RFTM-infection intensity (*r* = 0.64, value of *p* < 0.001).

### 3.2 Prevalence and infection intensity of *Perkinsus olseni* and *Perkinsus chesapeaki* at the spatial scale: single-and co-infections

#### 3.2.1 Diagnosis of *Perkinsus* in whole body samples

Global prevalence and infection intensities of single- and co-infected individuals across the Arcachon Bay were assessed by analysing the whole body of Manila clams using duplex qPCR methodology. In this study, a clam is qualified as “co-infected” when *P. olseni* and *P. chesapeaki* infect at least one of the six organs analysed.

The global prevalence of *Perkinsus* spp. in the whole body was 82% for Lanton and Gujan, 84% for Andernos, 90% for Piquey, and 96% for IAO (Figure 1A). Single-infection by *P. olseni* was detected in every station with prevalence ranging from 46% in Gujan (*n* = 23/50 clams) to 84% (*n* = 42/50 clams) in Andernos. Co-infection was detected in all stations except Andernos, with prevalence ranging from 12% (*n* = 6/50 clams) in Lanton to 36% (*n* = 17/50 clams) in Piquey. Surprisingly, single-infection by *P. chesapeaki* was rarely detected, with prevalence ranging from 2% (= 1/50 clams) in IAO to 14% (= 7/50 clams) in Gujan.

Infection intensities detected in the whole body showed differences particularly in *P. olseni*-single infections (Figure 1B). Samples from IAO had the highest mean intensity of infection with 8.5 log-nb. of copies. g^−1^ of wet tissue, whereas four other stations had mean intensity of infection varying from 7.2 log-nb. of copies. g^−1^ of wet tissue in Gujan to 8.4 log-nb. of copies. g^−1^ of wet tissue in Andernos (Wilcoxon *rank sum* test: value of *p* < 0.05; groups a and b; Figure 1B). For co-infected hosts, infection intensities between stations showed no difference (Wilcoxon *rank sum* test: value of *p* > 0.05; Figure 1B). The *P. chesapeaki*-single infection was of very low infection intensity with a mean of 3.8 log-nb. of copies. g^−1^ of wet tissue (i.e., 7.92 × 10^3^ copy number.g^−1^ of wet tissue) in Gujan (Supplementary Table S3).

#### 3.2.2 Diagnosis of BRD and BMD in Arcachon Bay

The clams were visually inspected during dissection to record the presence of symptoms associated with other diseases potentially detected in Arcachon Bay, such as BRD and BMD (Figure 1A). Brown organic conchiolin deposits classically identified as a BRD phenotype were only detected in Gujan and IAO with very low prevalence, 2 and 4%, respectively. The presence of necropsies of the posterior adductor muscle identified as a BMD phenotype was retrieved in all sampling sites with low to moderate prevalence ranging from 2% in Piquey to 14% in Gujan.

#### 3.2.3 Spatial patterns of *Perkinsus* spp. infection

**Figure 2:**
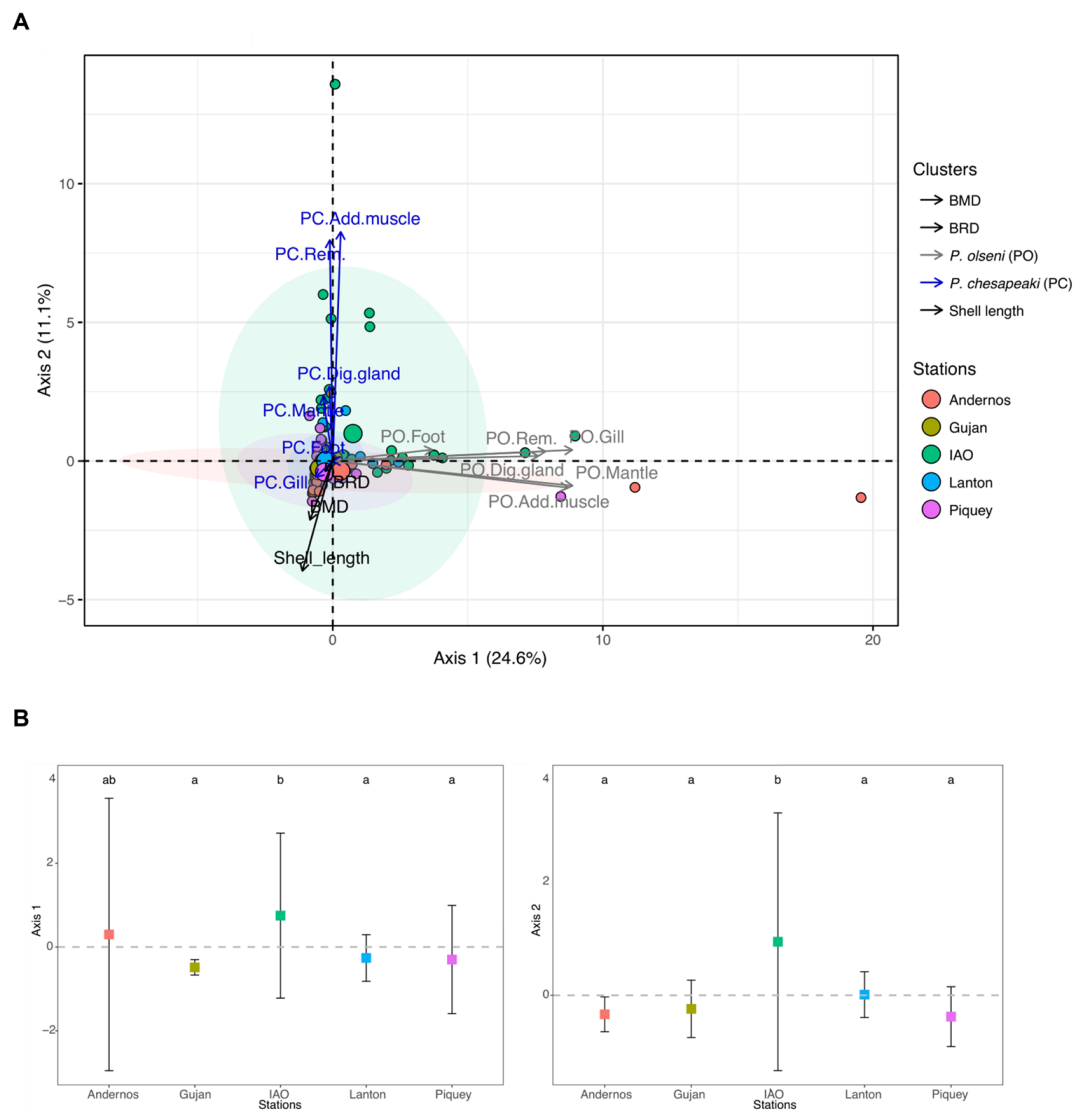
Principal component analysis (PCA) of *P. olseni* and *P. chesapeaki* infection intensities in different Manila clam organs from Arcachon Bay in November 2018. The first axis explains 24.6% of total variance of infection intensities while axis 2 explains 11.1%. **(A)** PCA including 250 individuals coloured by station. Ellipses included at least 50% of the individuals in a station. Variables implied in the PCA are the shell length, BMD, BRD and infection intensities specified by ‘PO’ for *P. olseni* and ‘PC’ for *P. chesapeaki* followed by the type of organ. Dig. gland, digestive gland; Add. muscle, adductor muscle; Rem. tissue, remaining tissue. **(B)** Individual values grouped by station on the Axis 1 and 2 of the PCA represented by infection intensities. Differences between stations were indicated by letters a, b and c according to multiple pairwise comparison of Dunn post-hoc test (value of *p* < 0.05).

**Figure 3:**
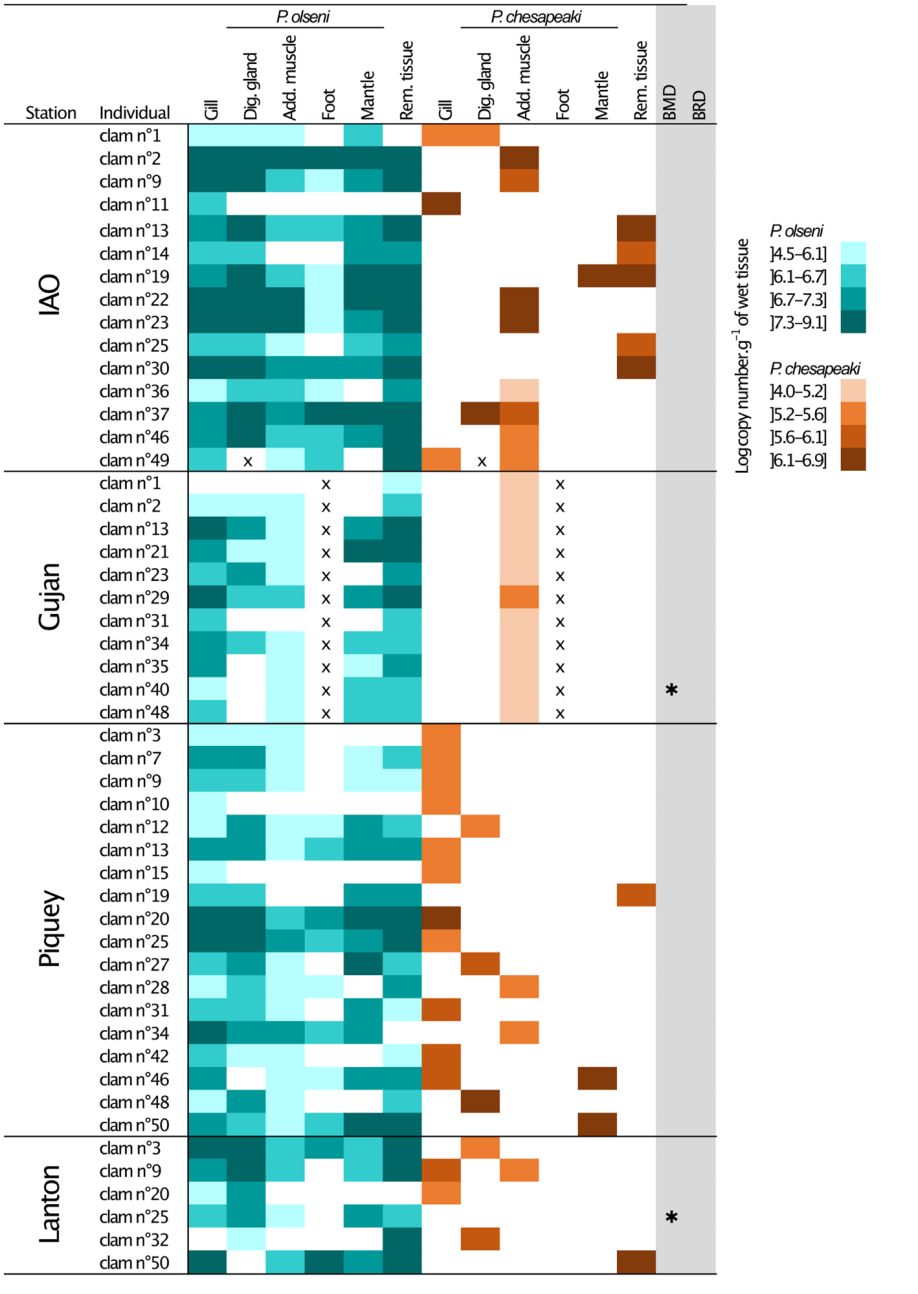
Infection intensities of co-infected individual clam per organs determined by qPCR duplex assays. The *P. olseni* repartition is represented in blue and the *P. chesapeaki* repartition in orange. Four levels of infection intensities were determined. x, missing value; Dig. gland, Digestive gland; Add. muscle, Adductor muscle; Rem. tissue, Remaining tissue; IAO, Ile aux Oiseaux; *, occurrence of Brown Muscle Disease (BMD) or Brown Ring Disease (BRD).

The results of the principal component analysis (PCA) performed on intensity of infection of *Perkinsus* species are presented in Figure 2. The axis 1, representing 24.6% of the total variance, was mainly explained by *P. olseni* infection intensities within the different organs, with contribution ranging from ~3.7% for foot to ~21.3% for mantle and gill (Figure 2A). The axis 2, representing 11.1% of the total variance, was mainly explained by *P. chesapeaki* infection intensities in all organs except gills. Indeed, the contribution of each organ ranged from ~0.1% to ~40.8% for foot to adductor muscle, respectively (Figure 2A). The ellipses of all sampling stations were not well separated along both axes (Figure 2A). Along axis 1, the intensity of infection by *P. olseni* in IAO sampling station was significantly different from those at Gujan, Lanton and Piquey stations. In addition, along the axis 2, the intensity of infection by *P. chesapeaki* in IAO sampling station was significantly different from those of other sampling stations (Figure 2B; Dunn post-hoc test: value of *p* < 0.01). Individual clams from Piquey station were characterised by high shell length (35.3 ± 2.7 mm; Tukey multiple comparison means test: value of *p* < 0.001) and the occurrence of *P. chesapeaki* in gill tissues (Figure 3 and Supplementary Figure S2). Other stations were characterised by smaller individual clam shell lengths (IAO 28.2 ± 2.1 mm and Lanton 29.3 ± 2.3 mm, compared to Andernos 31.9 ± 2.6 mm and Gujan 31.8 ± 5.27 mm; Tukey multiple comparison means test: value of *p* < 0.001) and *P. chesapeaki* being mainly localised in the adductor muscle (Figure 3 and Supplementary Figure S2).

### 3.3 Patterns of co-infection between *Perkinsus* species

Distribution of *P. olseni* and *P. chesapeaki* in each Manila clam organ was investigated to more accurately evaluate potential patterns in co-infected clams. In this study, clams were considered to be co-infected when both parasites are detected in at least one of the six organs (*n* = 50 co-infected individuals dispatched in four stations).

*Perkinsus olseni* appeared to be distributed in all organs tested with high infection intensity reaching up to 9.1 log-nb. of copies. g^−1^ of wet tissue (Figures 1B, 3). In co-infected hosts, *P. olseni* reached a maximum of 8.3 log-nb. of copies. g^−1^ of wet tissue (Figure 3). Conversely, *P. chesapeaki* was more heterogeneously distributed across organs, with weaker infection intensity reaching 6.9 log-nb. of copies. g^−1^ of wet tissue. Our results highlighted four different profiles between the five sampling stations: (1) co-infection was often detected in gill tissue and characterised by medium *P. chesapeaki* infection intensity as, for example, in Piquey (between 5.2 and 5.6 log-nb. of copies. g^−1^ of wet tissue, *n* = 11/18 clams); (2) co-infection was more frequently detected in the adductor muscle (53% of co-infected Manila clams; *n* = 8/15 clams in IAO) with moderate infection intensity (5.6 to 6.9 log-nb. of copies. g^−1^ of wet tissue, *n* = 8/15 clams); (3) co-infection exclusively detected in the adductor muscle with low infection intensity (4.0 to 5.6 log-nb. of copies. g^−1^ of wet tissue, *n* = 11/11 clams in Gujan); and (4) co-infection was sporadically detected in the digestive gland, mantle or remaining tissue, dispatched across stations.

**Figure 4:**
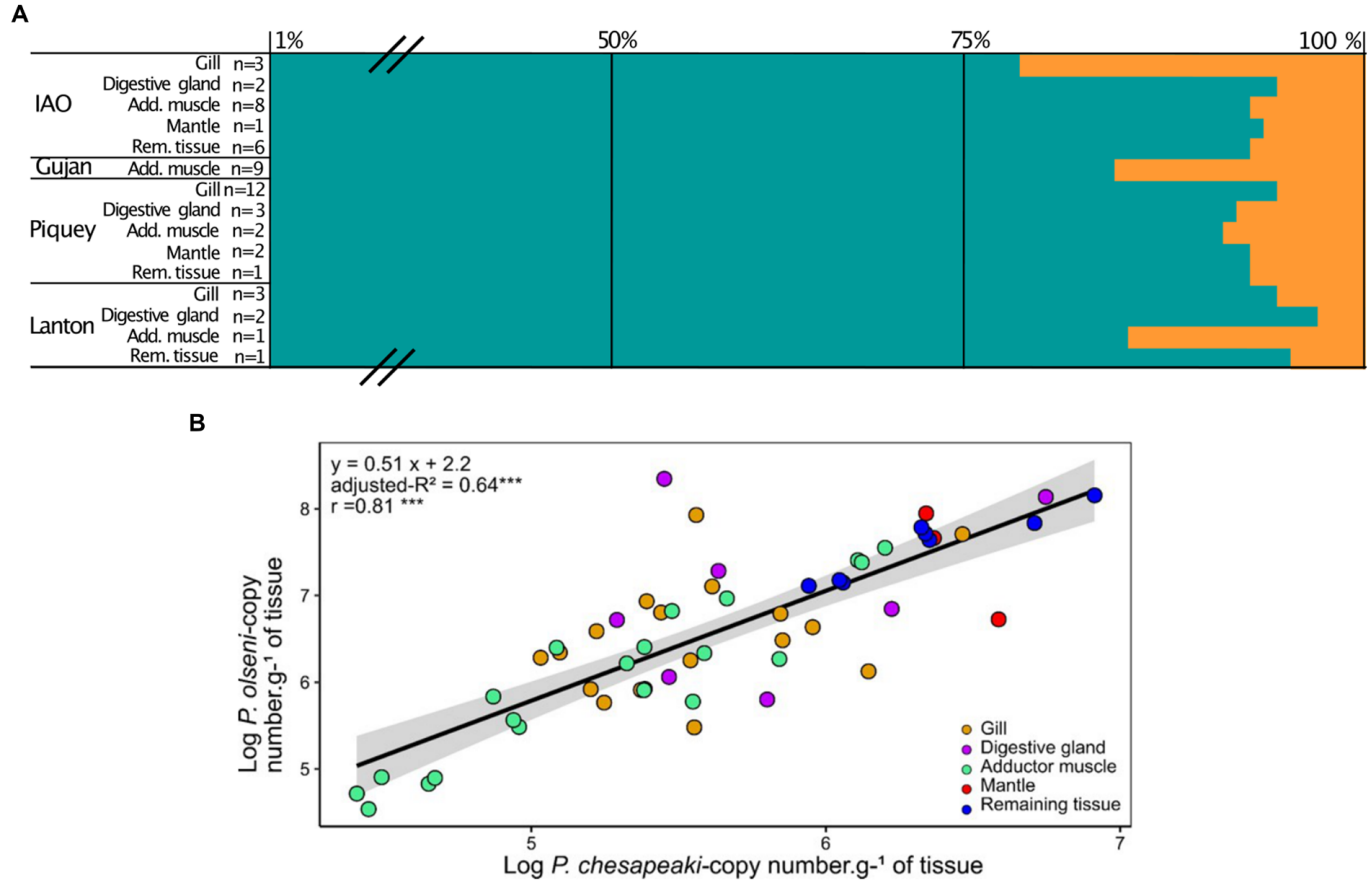
**(A)** Mean proportions of *P. olseni* (blue) and *P. chesapeaki* (orange) in common co-infected organ. Proportions are represented by the percentage of the mean of each parasitic species related to the total of copies of parasites in the organ. n: the number of clams co-infected by *P. olseni* and *P. chesapeaki*. Means and standard-deviation are detailed in Supplementary Table S4. Add. muscle, Adductor muscle; Rem. tissue, Remaining tissue. Means and standard deviations are detailed in Supplementary Table S5. **(B)** Linear regression between *P. olseni* and *P. chesapeaki* infection intensities (copy number.g^−1^ of wet tissue) from co-infected organ samples (*n* = 50). The linear relationship corresponds to the equation model: *y* = 0.51 x + 2.2 and explains 64% (adjusted-*R*^2^) of the variability. The relationship Organs (gill, digestive gland, adductor muscle, mantle and remaining tissue) were represented with coloured circles. r, Pearson’s coefficient. ‘***’ value of *p* < 0.001.

Proportions of *P. olseni* and *P. chesapeaki* in different compartments (gills, digestive gland, adductor muscle, mantle and remaining tissue) were assessed based on mean infection intensity (nb of copy number. g^−1^ of wet tissue) in order to estimate their spatial distribution within host tissues (Figure 4A and Table 2). We showed that *P. olseni* dominated all co-infected organs with a mean proportion of 92 ± 6%. For *P. chesapeaki*, the mean proportion was estimated at 8 ± 6%. In gill tissues, the weakest proportions of *P. olseni* were detected mainly in IAO (*n* = 3), with 77% of occupation while it was up to 96% for Piquey (*n* = 12) and Lanton (*n* = 3). In adductor muscle, the lowest proportions of *P. olseni* were detected in Gujan (*n* = 9) and Lanton (*n* = 1), with 84 and 85%, respectively. In every other organ (digestive gland, mantle and remaining tissue), *P. olseni* distribution took up over 90% of the total space (*n* = 18).

**Table 2:** Mean infection intensities of *P. olseni* and *P. chesapeaki* in common co-infected organs.

			Mean infection intensities (nb. of copies.g^−1^ of wet tissue)	
		*n*	*P. olseni*	*P. chesapeaki*
IAO	Gill	3	2.02×10^6^ ± 1.63×10^6^	6.02×10^5^ ± 6.89×10^5^
	Digestive gland	2	6.90×10^7^	2.94×10^6^
	Add. muscle	8	1.28×10^7^ ± 1.36×10^7^	7.45×10^5^ ± 5.75×10^5^
	Mantle	1	4.60×10^7^	2.33×10^6^
	Rem. tissue	6	5.60×10^7^ ± 4.76×10^7^	3.33×10^6^ ± 2.79×10^6^
Gujan	Add. muscle	9	3.69×10^5^ ± 5.27×10^5^	7.10×10^4^ ± 5.81×10^4^
Piquey	Gill	12	1.41×10^7^ ± 2.62×10^7^	6.12×10^5^ ± 7.64×10^5^
	Digestive gland	3	1.05×10^7^ ± 7.60×10^6^	7.67×10^5^ ± 7.94×10^5^
	Add. muscle	2	3.61×10^6^	3.27×10^5^
	Mantle	2	4.69×10^7^	3.03×10^6^
	Rem. tissue	1	1.29×10^7^	8.76×10^5^
Lanton	Gill	3	5.25×10^6^ ± 6.51×10^6^	2.32×10^5^ ± 1.56×10^5^
	Digestive gland	2	1.11×10^8^	4.58×10^5^
	Add. muscle	1	2.17×10^6^	3.87×10^5^
	Rem. tissue	1	6.10×10^7^	2.11×10^6^

Means and standard-deviation (sd) are in number of copies per gram of wet tissue samples (Mean ± sd). n: the number of individual organs infected by *P. olseni* and *P. chesapeaki*. Add. muscle, Adductor muscle; Rem. tissue, Remaining tissue.

At the Gujan sampling station, *P. chesapeaki* was only detected in the adductor muscle (Figure 3). Due to technical issues, we were not able to weigh foot samples before molecular analysis. The intensity of infection for these samples is thus missing only for this station. However, no co-infection has been detected in foot samples in other individual clams from other stations, we can therefore assume that this trend might be similar for *P. chesapeaki* in Gujan.

To understand the distribution of co-infection across tissues, infection intensities of *P. olseni* and *P. chesapeaki* in co-infected organ samples were plotted (Figure 4B). A linear regression associated both parasitic species (*y* = 0.51x + 2.2, adjusted-*R*^2^ = 0.64, *n* = 50 of co-infected organs). A significant Pearson’s coefficient showed a strong positive correlation between *P. olseni* and *P. chesapeaki* infection intensities (*r* = 0.81, value of *p* < 0.001). Mantle (*n* = 3) and remaining tissue (*n* = 8) samples showed the highest infection intensities of *P. olseni* (> 6.7 log-nb. of copies. g^−1^ of wet tissue. g^−1^ of wet tissue) and *P. chesapeaki* (> 5.6 log-nb. of copies. g^−1^ of wet tissue). Gill, digestive gland and adductor muscle samples did not aggregate following infection level. However, low values of *P. olseni* (< 6.1 log-nb. of copies. g^−1^ of wet tissue) and *P. chesapeaki* (< 5.2 log-nb. of copies. g^−1^ of wet tissue) were mainly found in the adductor muscle (*n* = 8) (Figure 4B). This relationship revealed that co-occurrence of both parasitic species within the same organ, concerning 96% of co-infected hosts (*n* = 48/50 co-infected clam), occurred in a restricted range of infection intensity from 4.5 and 6.9 log-nb. of copies. g^−1^ of wet tissue. Above this threshold, only *P. olseni* single-infection and co-infection in different organs (*n* = 2) were observed.

## 4 Discussion

The development of the duplex qPCR methodology allows the quantification of infection intensity and prevalence of both parasitic species, *P. olseni* and *P. chesapeaki,* in each organ of Manila clams from populations of the Arcachon Bay, as described by Itoïz et al. (2021) (Figure 1 and Supplementary Figure S1). The concordance parameter (81.6 ± 5.2%) and the linear regression (*y* = 0.61x + 4.34, *R*^2^ = 0.41; Spearman correlation coefficient: *r* = 0.64, value of *p* < 0.001) strengthen the reliability of this molecular method to assess prevalence of *Perkinsus* species from *in situ* samples. In this study, we decided to discard samples with Ct values below the quantifiable range (2.1×10^1^ total copies), thus underestimating the number of low infection intensities that could however be quantified using the RFTM methodology. Indeed, we observed that 14% of samples (*n* = 36 / 250 gill samples) were negative to qPCR assays but positive in RFTM assays, and conversely 4% of samples were positive to qPCR assays but negative in RFTM assays (*n* = 10/250 gill samples). One explanation could be the asymmetric distribution of the parasite within the two gills. Hence, these results lead to a slight to substantial agreement between both methodologies depending of sampling sites. That said, only qPCR assays allow for a distinction between *P. olseni* and *P. chesapeaki* within a tissue sample.

### 4.1 Single-infection and co-infection in Arcachon Bay

#### 4.1.1 At the spatial scale

The monitoring of single-infections and co-infections provides a better understanding of Perkinsosis in Arcachon Bay. In this study, stations are mainly differentiated following *P. olseni* infection intensities with: medium-low values in Gujan (0.13×10^8^ ± 0,28×10^8^ nb. of copies.g^−1^ of wet tissue) to high values in IAO and Andernos (3.06×10^8^ ± 3.91×10^8^ and 2.60×10^8^ ± 9.10×10^8^ nb. of copies.g^−1^ of wet tissue respectively) (Supplementary Table S3). Clams with high infection intensity are retrieved from the external station IAO and the internal station Andernos (Figure 1B). This distribution could be explained by environmental abiotic factors, such as lower salinity and muddy sediment in internal stations due to the stronger influence of continental freshwater input, or the higher salinity and medium sand composition found in external stations (Dang et al., 2010a; Binias et al., 2014). Indeed, Arcachon Bay is a peculiar ecosystem where marine and continental water inputs mix, resulting in varying temperature and salinity gradients between stations (Plus et al., 2006). Dang et al. (2013) demonstrated lower infection intensity close to the Leyre river mouth located between Gujan and Lanton sampling stations, which is coherent with our observations. Conversely, higher infection intensity and prevalence of *P. olseni* are expected to be correlated with size, the age of the clam or higher salinity levels with the optimal salinity range for *P. olseni* is between 25 and 35 (Auzoux-Bordenave et al., 1996; Park and Choi, 2001; Dang et al., 2010a). Hence, the higher salinity observed in oceanic stations may explain such dichotomy in our observations except for Piquey sampling station (Dang et al., 2010a; Binias et al., 2014). This station is influenced by oceanic currents but harboured lower infection intensities compared to IAO and Andernos which could be explained by its proximity to the mouth of the lagoon with a better accessibility to fresh input of oceanic phytoplankton (Dang et al., 2009, 2010a,b). Moreover, there is no clear distribution of co-infection between stations except for Piquey. Significant occurrences of *P. chesapeaki* in gills appear to depend on clam shell size, suggesting a higher primary infection of the parasite due to higher clearance activity. Despite this exception, *P. chesapeaki* seems here to be a secondary infection in Manila clams.

In this study, the prevalence of Brown muscle disease (BMD) and Brown ring disease (BRD) phenotypes were quite low compared to previous monitoring results in Arcachon Bay (Lassalle et al., 2007; Dang et al., 2008; Binias et al., 2014). However, our results show here that BMD and BRD, both of which can be responsible for Manila clam mortality (Dang et al., 2008), are not correlated with Perkinsosis distribution across sampling sites.

#### 4.1.2 At the individual scale

If we consider the analysis carried out on the whole Manila clam body, cases of single-infection by *P. olseni* are dominant in all stations and represented in all cases of infection detected at the Andernos station. Co-infection is regularly detected across Gujan, IAO, Lanton and Piquey, ranging from 12 to 36% of total infections. Conversely, *P. chesapeaki* single-infections are restricted in Gujan and IAO stations with very low prevalence, 14 and 2% respectively, combined with low infection intensities. It is common for one pathogen to influence the acquisition of and/or the infection dynamics of a second opportunistic pathogen (Susi et al., 2015). Two hypotheses could be formed: (1) *P. olseni* may play a facilitating role in the development of *P. chesapeaki* infections in the Manila clam. The settlement of a first parasite can be beneficial to a secondary parasite as the host is already immune-compromised by the initial infection; a gross probability calculation supports this hypothesis. Taking the example of IAO (Supplementary Table S4), the percentage of *P. olseni* single-infection is 64% and the percentage of *P. chesapeaki* single infection is 2%. In a case of random co-infection, the prevalence of double infection should be (64 × 2)/100 = 1.3%. However, the observed prevalence of co-infection was 30%. A similar trend was observed in the other stations, suggesting a facilitation process, but without knowing which parasite facilitates the infection process for the other. (2) *P. chesapeaki* infection may be independent of *P. olseni* infection. Indeed, in Europe, along the Galician coast (Spain), Ramilo et al. (2016), recorded a low number of co-infections between *P. chesapeaki* and *P. olseni.* They hypothesised an accidental or punctual introduction as well as a poor adaptation of *P. chesapeaki* to a “new” host. However, co-infection by multiple *Perkinsus* species is a recurrent phenomenon as described, for example, along East coast of United States, *P. marinus, P. chesapeaki* are detected co-infecting sympatric host bivalves (e.g., the baltic clam *Macoma balthica*, the soft-shell clam *Mya arenaria*, the oyster *C*rassostrea *virginica,* and the hard clams *Mercenaria mercenaria*) (Coss et al., 2001; Pecher et al., 2008; Reece et al., 2008; Marquis et al., 2020).

### 4.2 Co-infection: a patchy distribution at the individual scale

Among *Perkinsus* species, *P. marinus* is known to be phagocytosed and divided by oyster hemocytes, a process that plays a significant role in parasitic infection (La Peyre et al., 1995a,b; Villalba et al., 2004; Bassem et al., 2013; Lau et al., 2018). In contrast, in clams, while *P. olseni* and *P. chesapeaki* cells have been observed to be occasionally phagocytosed by hemocytes (e.g., Burreson et al., 2005), the question of their viability after phagocytosis remains unclear. Therefore, it is still uncertain whether these parasites propagate extracellularly or within hemocytes or both.

Among co-infected clams, *P. olseni* infection appears to be homogeneously distributed across all tissue samples, while the detection of *P. chesapeaki* is often restricted to one or two organs. We identified here three major detection patterns of co-infected clams: *P. chesapeaki* detection (1) is mainly prevalent in gill tissues in the Piquey station clams (*n* = 11/18 clams) ranging from 5.0 to 6.4 log-nb. of copies. g^−1^ of wet tissue; (2) is present in the adductor muscle mainly in Gujan (*n* = 11/11) and IAO (*n* = 8/15); (3) displays a moderate level of infection in the digestive gland, mantle and remaining tissue (5.9 to 6.9 log-nb. of copies. g^−1^ of wet tissue) distributed homogeneously across stations.

The high variability of co-infection groups, in terms of organ(s) or stations, suggests that *P. chesapeaki* may be more affected by local biotic or abiotic factors than *P. olseni*. Hence co-infection patterns may be the result of a complex overlap between individual infection level of the host and the presence of other micro or macro parasites or environmental factors affecting both the host and the parasites (e.g., sediment type, localisation in the basin, temperature, salinity). Finally, 98% of co-infected hosts are associated with moderate *P. olseni* infection intensities. A positive relationship links *P. olseni* and *P. chesapeaki* infection intensities when they are detected in sympatry within the same organ. However, this relationship theoretically demonstrates that we should not detect *P. chesapeaki* when the infection intensity of *P. olseni* falls below 2.2 log-nb. of copies. g^−1^ of wet tissue. Here, the recurrent observation that *P. olseni* dominates the co-infected organs of *R. philippinarum* is in accordance with the results obtained by Arzul et al. (2012) regarding *Ruditapes decussatus* in the Leucate lagoon (Mediterranean Sea). However, in the same study, *P. chesapeaki* was predominating in *R. philippinarum* at Bonne Anse (Atlantic coast). Such results support the previous hypothesis that *P. chesapeaki* infections may be facilitated by moderate infection of *P. olseni* which could increase transepithelial migration of hemocytes, as described for *P. marinus* in *C. virginica* (Lau et al., 2018), or reduce the efficiency of host defence mechanisms similarly to secondary infections by bacteria or virus (Montes et al., 2001). Hence, our results suggest a competitive exclusion of *P. chesapeaki* infection in cases of high *P. olseni* infection intensity due to direct or indirect interspecific competition for resource or space within host tissues.

Interestingly, in all sampling stations, *P. chesapeaki* was never detected in the foot. The possibility of a PCR amplification bias was excluded because *P. chesapeaki* was detected in artificial infection with a plasmid mix containing *P. chesapeaki* ITS sequence and host foot gDNA (Itoïz et al., 2021). The foot is one of the last organs to be infected by *P. olseni* when the infection intensity is heavy (Wang et al., 2018). Thus, if *P. chesapeaki* were a secondary invader as previously hypothesised, the detection of *P. chesapeaki* cells may correspond either to an early infection or to the parasite’s inability to infect the foot compartment the way *P. olseni* does. Sporadic detections of *P. chesapeaki* have also been confirmed by histology techniques in different *in situ* studies on co-infection (Reece et al., 2008; Arzul et al., 2012; Ramilo et al., 2016). *P. chesapeaki* was described in the digestive gland of two *R. philippinarum* individuals by Ramilo et al. (2016), while detection in the digestive gland and in gonadal tissue were also reported in *R. decussatus* (Arzul et al., 2012). In other bivalves, such as *Mya arenaria* or *Cyrtopleura costata*, infection in the gills was also detected but appeared localised and concentrated into compact cell clusters (Reece et al., 2008). Low throughout-tissue spreading and infrequent detection of infection in *Ruditapes* species maintains the hypothesis that these clams are a “sub-optimal” host for *P. chesapeaki*.

The variability in the kinds of organ infected by *P. chesapeaki* may confirm that local conditions can influence the co-infection process at a small scale. Surprisingly, Gujan station showed an unexpected *P. chesapeaki* infection pattern with only one organ infected, the adductor muscle. Dang et al. (2008) and Binias et al. (2014) hypothesised, in the case of BMD, that the posterior muscle, being located nearest to the sediment surface, was more vulnerable to certain pathogenic agents and environmental variations. Even if, in this study, BMD and BRD distributions were not correlated to *Perkinsus* distributions, the sediment may still represent a potential reservoir of *Perkinsus* spp. (Choi, 2002; Park et al., 2010). Thus, muddy sediments may better retain *Perkinsus* hypnospores (Binias et al., 2014) and favour contamination by this life stage.

## 5 Conclusion

The recent detection of two *Perkinsus* species highlights recurrent cryptic infections in host clam populations. In this study, *Perkinsus* infections are dominated by *P. olseni* and appear along an environmental gradient corresponding to its optimal proliferation conditions. Conversely, *P. chesapeaki* is recurrent in Manila clams across five sampling stations with moderate and low prevalence in co-infection and in single-infection, respectively, suggesting a more secondary role of *P. chesapeaki* infection. Moreover, the organ-targeted infection pattern seen with *P. chesapeaki* compared to more widespread whole-body infections by *P. olseni* supports the hypothesis that *R. philippinarum* may not be the preferred host of *P. chesapeaki*. To date, it is not possible to conclude whether *P. olseni* facilitates the opportunistic infection of *P. chesapeaki* or whether this secondary infection is independent. Taken all together, these data support the hypothesis that *P. chesapeaki* was introduced in Europe alongside other bivalves’ species, *Mya arenaria* or *Mercenaria mercenaria*, and confirms the need to monitor these parasites, which are able to spill over to new indigenous host populations, using a combination of histological and molecular analyses. It is now urgent and timely to expand our view of the host range of *Perkinsus* species and investigate the sympatric benthic fauna for hot-spots of co-infections. In the light of these results, the co-infection process is a Pandora’s box, justifying the need to shift from the study of isolated pathogens to a more integrated approach.

## Data availability statement

The original contributions presented in the study are included in the article/Supplementary material, further inquiries can be directed to the corresponding authors.

## Author contributions

SI, PS, and AC designed the study. SI, CM, and MP performed the analyses. AC provided the funds. All authors drafted the article and approved the final version of the manuscript.
