# Supplementary material for: Co-infection of two eukaryotic pathogens within clam populations in Arcachon Bay
Source: Front Microbiol. 2024 Jan 8;14:1250947. doi: 10.3389/fmicb.2023.1250947 (PMC10800547; doi:10.3389/fmicb.2023.1250947)

**Figure S1.** Linear regressions among Ct values, infection intensities determined by RFTM and by qPCR assays. The infection intensities determined by RFTM are based on the global counting of *Perkinsus* hypnospores (nb. of cells.g^-1^ of wet gill) while the intensity of infection determined using qPCR assays was based on the sum of *P. olseni* and *P. chesapeaki* copies (nb. of copies.g^-1^ of wet gill). RFTM and qPCR assays relationship: y=-0.56x+4.34. Ct: Cycle threshold value. r: Pearson’s coefficient. '***' p-value <0.001.

**Figure S2.** Shell length of Manila clams sampled Arcachon bay in 2018. Stations were compared each other. Shell length variable is normally distributed (Shapiro-Wilk normality test: W = 0.99, p-value = 0.2). Analysis of variance (ANOVA) demonstrates significant difference between station (p-value < 0.001). A Tukey multiple comparisons of means test (95% family-wise confidence level) were realised to determine significant difference between stations depending on shell length of Manila clam. Differences are represented by a, b and c.


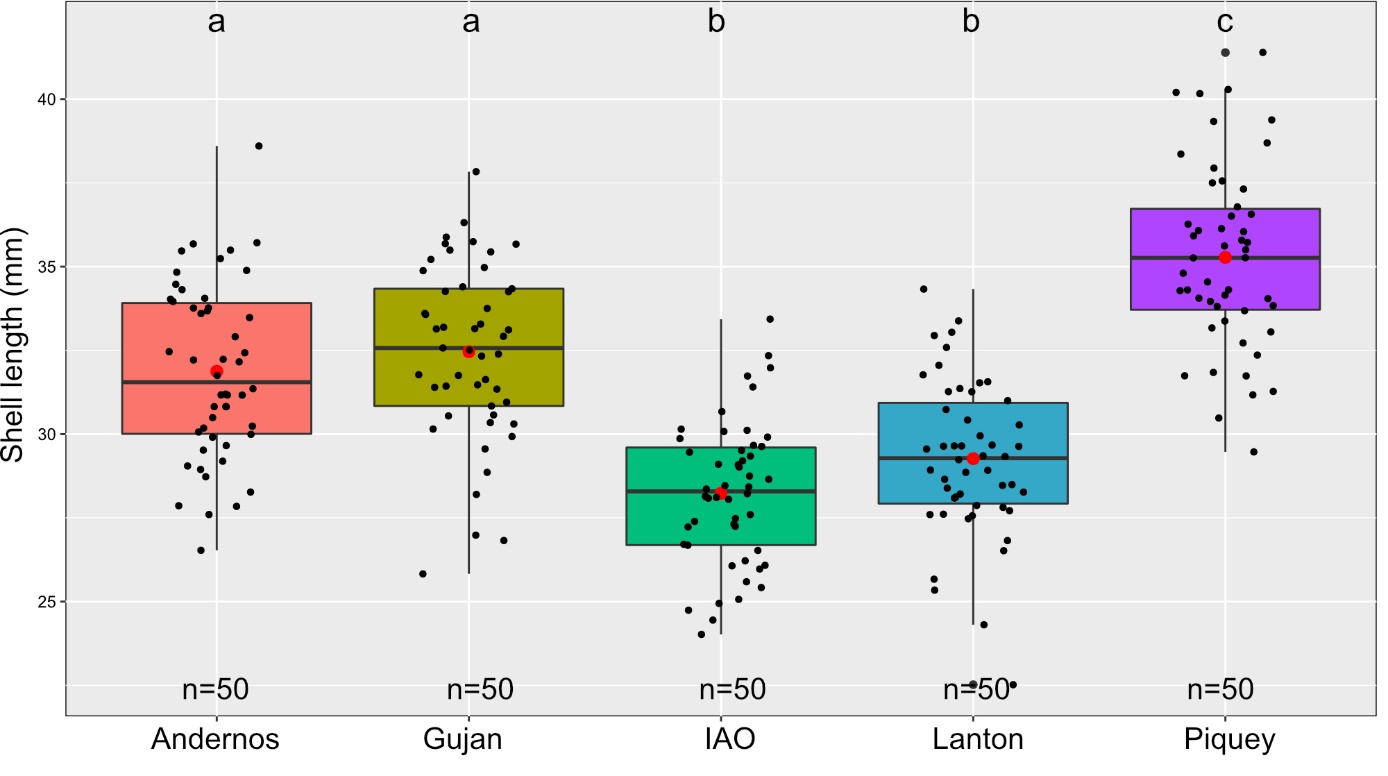

Supplement: Supplementary file 1 [file Data_Sheet_1.DOCX]
