# Supplementary material for: Co-infection of two eukaryotic pathogens within clam populations in Arcachon Bay
Source: Front Microbiol. 2024 Jan 8;14:1250947. doi: 10.3389/fmicb.2023.1250947 (PMC10800547; doi:10.3389/fmicb.2023.1250947)
Supplement: Supplementary file 2 [file Data_Sheet_2.DOCX]

**Table S1.** Characteristics of the five sampling stations: latitude, longitude, salinity, grain-size median (µm), silt & clay content (% of dry weight sediment), organic matter content (% of dry weight sediment), tidal level (m).

| Site | Latitude | Longitude | Salinity | Grain size median | Silt & clay content | Organic matter content | Tidal level |
| --- | --- | --- | --- | --- | --- | --- | --- |
| Andernos^1^ | 44°42'31"N | 1°8'11"W | 29.9 | 163 | 14.54 | 3.33 | 2.11 |
| Gujan^1^ | 44°39'59" N | 1°5'29" W | 29.9 | 69 | 47.17 | 5.63 | 1.46 |
| IAO^1^ | 44°41'60" N | 1°10'1" W | 32.8 | 97 | 42.45 | 13.05 | 2.63 |
| Lanton^1^ | 44°41'31" N | 1°4'48" W | 29.9 | 78 | 40.99 | 10.09 | 1.89 |
| Piquey^2^ | 44°41'11" N | 1°12'55" W | 32.8 | 112 | 32.62 | 6.65 | 1.45 |

^1^ Dang et al, 2010b (except salinity^2^)

^2^ Binias et al. 2014 and personal data

**Table S2.** Concordance, discordance and Cohen's Kappa (κ) coefficient between RFTM and qPCR assays on gill tissue samples.

| Station | Discordance (%) | Concordance (%) | κ |
| --- | --- | --- | --- |
| IAO | 14 | 86 | 0.41 |
| Andernos | 24 | 76 | 0.35 |
| Gujan | 24 | 76 | 0,42 |
| Lanton | 14 | 86 | 0.62 |
| Piquey | 16 | 84 | 0.17 |

**Table S3.** Mean infection intensities of single-*P. olseni* (PO single), single-*P. chesapeaki* (PC single) infections and co-infection (Co-infection) in whole clam body evaluated by qPCR assays from five sampling station. Means and standard-deviation (sd) are in number of copies per gram of wet tissue samples (Mean ± sd). n in bracket correspond to the number of clam infected for each condition.

|  | Whole Body mean infection intensities  (nb. of copies.g^-1^ of wet tissue) | | |
| --- | --- | --- | --- |
|  | PO single-infection | PC single-infection | Co-infection |
| IAO | 3,06x10^8^ ± 3,91x10^8^ (n=32) | 3,06x10^8^ (n=1) | 4,65x10^7^ ± 6,32x10^7^ (n=15) |
| Gujan | 1,76x10^7^ ± 2,82x10^7^ (n=23) | 7,92x10^3^ ± 5,07x10^3^ (n=7) | 2,37x10^7^ ± 4,35x10^7^ (n=11) |
| Lanton | 6,28x10^7^ ± 1,79x10^8^ (n=35) | 0 | 3,21x10^7^ ± 3,53x10^7^ (n=6) |
| Piquey | 7,98x10^7^ ± 2,20x10^8^ (n=27) | 0 | 1,06x10^7^ ± 1,76x10^7^ (n=27) |
| Andernos | 2,60x10^8^ ± 9,10x10^8^ (n=42) | 0 | 0 |

**Table S4.** Prevalence of *Perkinsus* spp., single-*P. olseni* (PO single), single-*P. chesapeaki* (PC single) infections and co-infection (Co-infection) in whole clam body evaluated by qPCR assays from five sampling station in November 2018 in Arcachon Bay. Prevalences are in '%'.

|  | *Perkinsus* spp. | PO single | PC single | Co-infection |
| --- | --- | --- | --- | --- |
| IAO | 96 | 64 | 2 | 30 |
| Gujan | 82 | 46 | 14 | 22 |
| Lanton | 82 | 70 | 0 | 12 |
| Piquey | 90 | 54 | 0 | 36 |
| Andernos | 84 | 84 | 0 | 0 |
